# Case Report: Invasive Pentastomes, *Raillietiella orientalis* (Sambon, 1922), in a Free-Ranging Banded Water Snake (*Nerodia fasciata*) in North Central Florida, USA

**DOI:** 10.3389/fvets.2020.00467

**Published:** 2020-08-04

**Authors:** Heather D. S. Walden, Marley E. Iredale, April Childress, James F. X. Wellehan, Robert J. Ossiboff

**Affiliations:** Department of Comparative, Diagnostic and Population Medicine, University of Florida College of Veterinary Medicine, Gainesville, FL, United States

**Keywords:** Burmese python, colubrid, parasite, Pentastomida, pentastomiasis, *Raillietiella orientalis*, water snake

## Abstract

*Raillietiella orientalis* is an obligate, crustacean parasite that resides in the respiratory tract of definitive snake hosts. Common throughout southeastern Asia and Australia, *R. orientalis* is believed to have been introduced into southern Florida, United States along with Burmese pythons (*Python bivittatus*) in the 1990s. While the invasive range of Burmese pythons is restricted to southern Florida, *R. orientalis* has advanced north in the state in native snake species. *R. orientalis* were recovered from the lungs, trachea, oral cavity, and esophagus of an emaciated adult female free-ranging banded water snake (*Nerodia fasciata*) in north central (Alachua County), Florida, USA. Concurrent findings included the recovery of *Ochetosoma* sp. trematodes from the oral cavity, and multifocal dermal lesions consistent with snake fungal disease (*Ophidiomyces ophiodiicola*). This is the first report of *R. orientalis* in north central Florida, well outside the invasive range of the Burmese python, documenting the substantial northward expansion of the known geographical range of this invasive pentastome in Florida.

## Introduction

Pentastomes are obligate, internal parasites found mainly in the respiratory tracts of their definitive hosts, which include mammals, reptiles, amphibians and birds. Pentastomes have indirect life cycles, and use mammals, reptiles, amphibians, fish, or coprophagous insects as intermediate hosts (1). Large eggs are found in the feces of the definitive host; when ova are mature, they contain a larva which will develop in the tissues of the intermediate host. Nymphal stages are ingested by the definitive host, where they develop to an adult. The anterior ends consist of four chitinized hooks and a buccal cadre that allow the parasite to attach to the host and feed on blood (1). Pentastome species inhabit the lungs of reptiles more than any other host clade, and the openness of the reptile lung may make it an easy niche to populate (2). These parasites can become very large (up to 10 cm long) in the host lung. Morbidity and mortality due to pentastome infections in reptiles are usually associated with pneumonia. Migration of larval and nymphal stages can cause disease in definitive, intermediate, and aberrant hosts (1).

**Figure 1:**
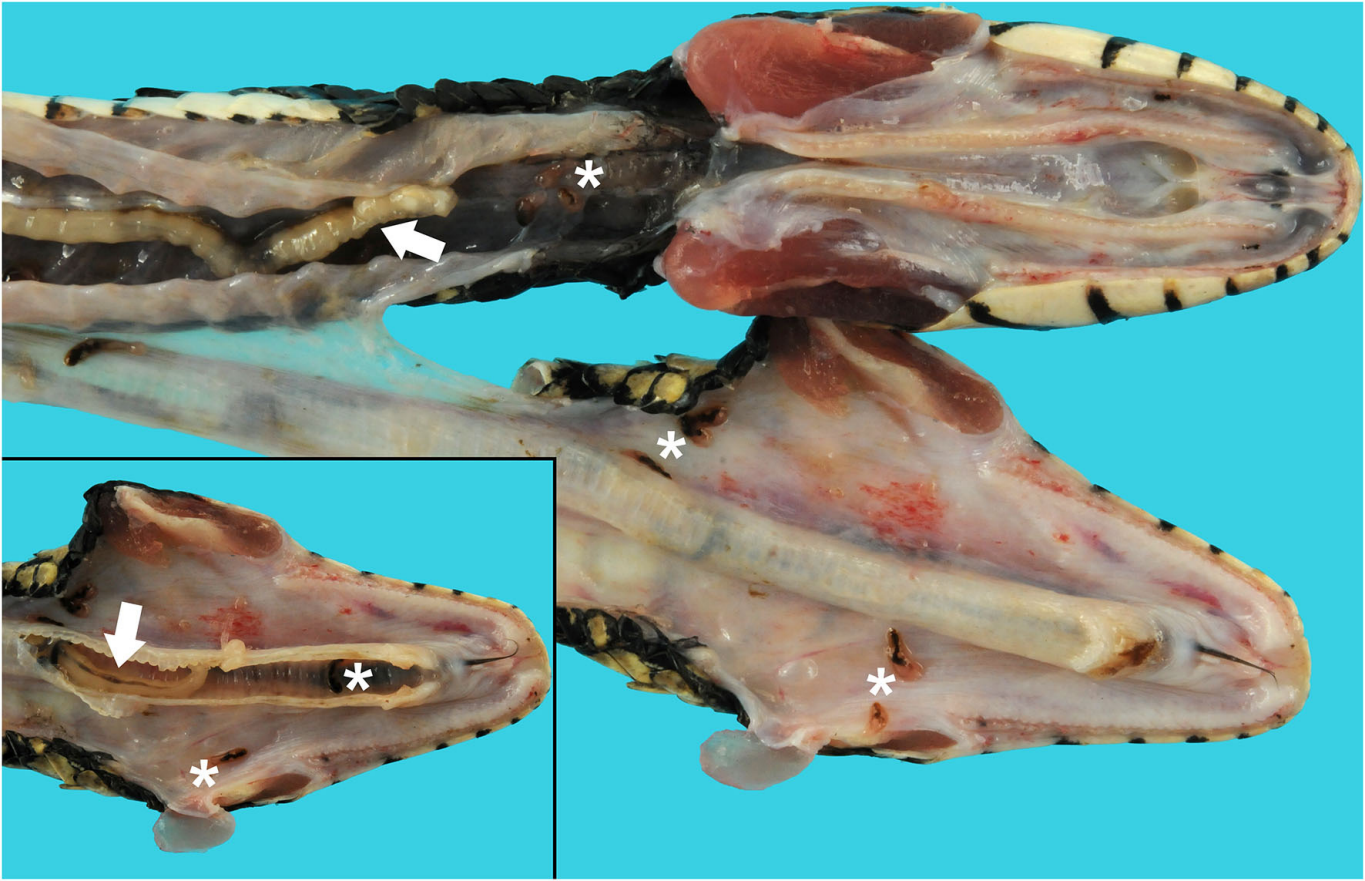
Tracheal and esophageal pentastomes and trematodes in a banded water snake (*Nerodia fasciata*). The jaw is unilaterally disarticulated and positioned laterally to expose the oral cavity, glottis, and esophagus. *Raillietiella orientalis* pentastomes (white arrows) and *Ochetosoma* sp. trematodes (white asterisks) are present in the oral cavity, esophagus, and lumen of the trachea (inset).

An adult, female, free-ranging banded water snake (*Nerodia fasciata*) was found emaciated, extremely weak, and with no righting reflex after being eaten and regurgitated by another snake [a black racer (*Coluber constrictor*)] in Alachua, County, Florida in May of 2019. The snake was treated with fluids, antibiotics, and supportive care. During hospitalization, fecal flotation revealed pentastome ova; the snake was found dead on day 4 before the planned endoscopic exam and removal of adult pentastomes could be performed. A complete postmortem examination was performed. On gross necropsy, numerous adult metazoan parasites were recovered from the oral cavity, esophagus, and respiratory tract (Figure 1). In the oral cavity and choana, there were six adult pentastomes and approximately seven dark black-red trematodes (renifers). In the esophagus there were three adult pentastomes and approximately seven trematodes (Figure 1). In the trachea there were three adult pentastomes (Figure 1, inset), and in the lungs there were approximately five adult pentastomes. There were also five adult pentastomes in the connective tissue ventral to the spine and dorsal to the liver. There were multifocal dark brown ovoid discolorations of the ventral scales, measuring 0.3–0.5 cm in diameter, that were associated with roughening of the scale margins distributed along the length of the ventrum.

**Figure 2:**
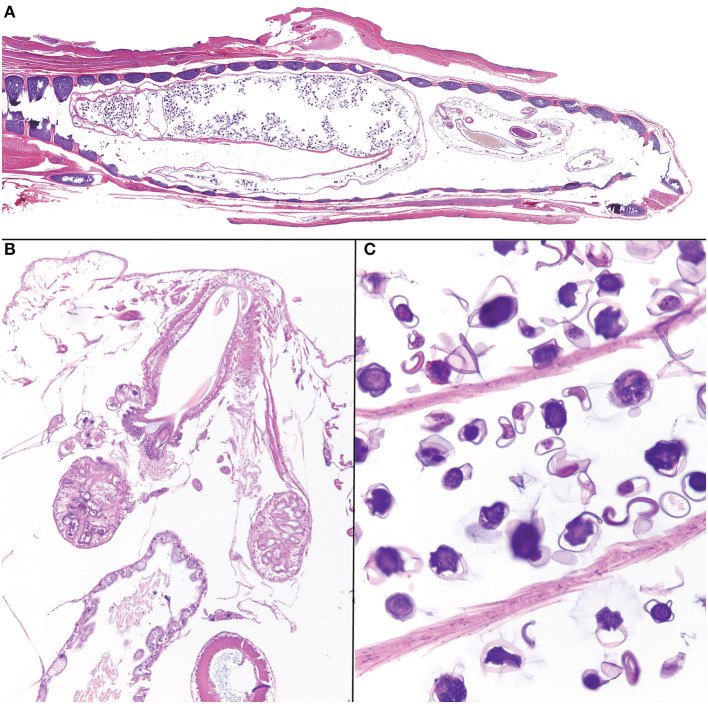
Photomicrographs of *Raillietiella orientalis* pentastomes within the trachea of a banded water snake (*Nerodia fasciata*). **(A)** On subgross examination, cross and longitudinal male and female parasites fill the lumen of the trachea. **(B)** The buccal cadre of a male parasite with hooks and acidophilic glands. **(C)** Ova within the lumen of a female *Raillietiella orientalis*.

Histologically, within the lumen of the glottis/trachea there were multiple longitudinal and transverse sections of pentastome parasites (Figure 2A). Male pentastomes measured up to 1,500 microns in diameter, and were characterized by the presence of a buccal cadre and hooks (Figure 2B), acidophilic glands (Figure 2B), a digestive tract lined by ciliated uninucleate enterocytes and containing brown-orange pigment (digested blood; Figure 2B), and an eosinophilic cuticle with refractile, round spiracles. Female pentastomes measured up to 2,250 microns in diameter, and sections were dominated by a reproductive tract filled with dozens of 50–100 micron diameter ovoid ova with a translucent, slightly refractile shell (Figure 2C). The trachea itself had mild, multifocal tracheal epithelial attenuation and heterophilic to mucoid tracheitis with intraluminal pentastome ova. Few granulomas with central cores of eosinophilic debris and slightly refractile, translucent, yellow material were also present adjacent to the trachea and deep to the oral mucosa. Other concurrent systemic findings included bacterial bronchopneumonia, chronic necrotizing and granulomatous hepatitis, and generalized cutaneous and facial dermatitis with intralesional fungal hyphae and arthroconidia. PCR of formalin-fixed paraffin embedded ribbons of affected skin were confirmed to contain *Ophidiomyces ophiodiicola* by quantitative PCR by the Zoological Medicine Diagnostic Laboratory, College of Veterinary Medicine, University of Florida, confirming a diagnosis of ophidiomycosis (snake fungal disease).

**Figure 3:**
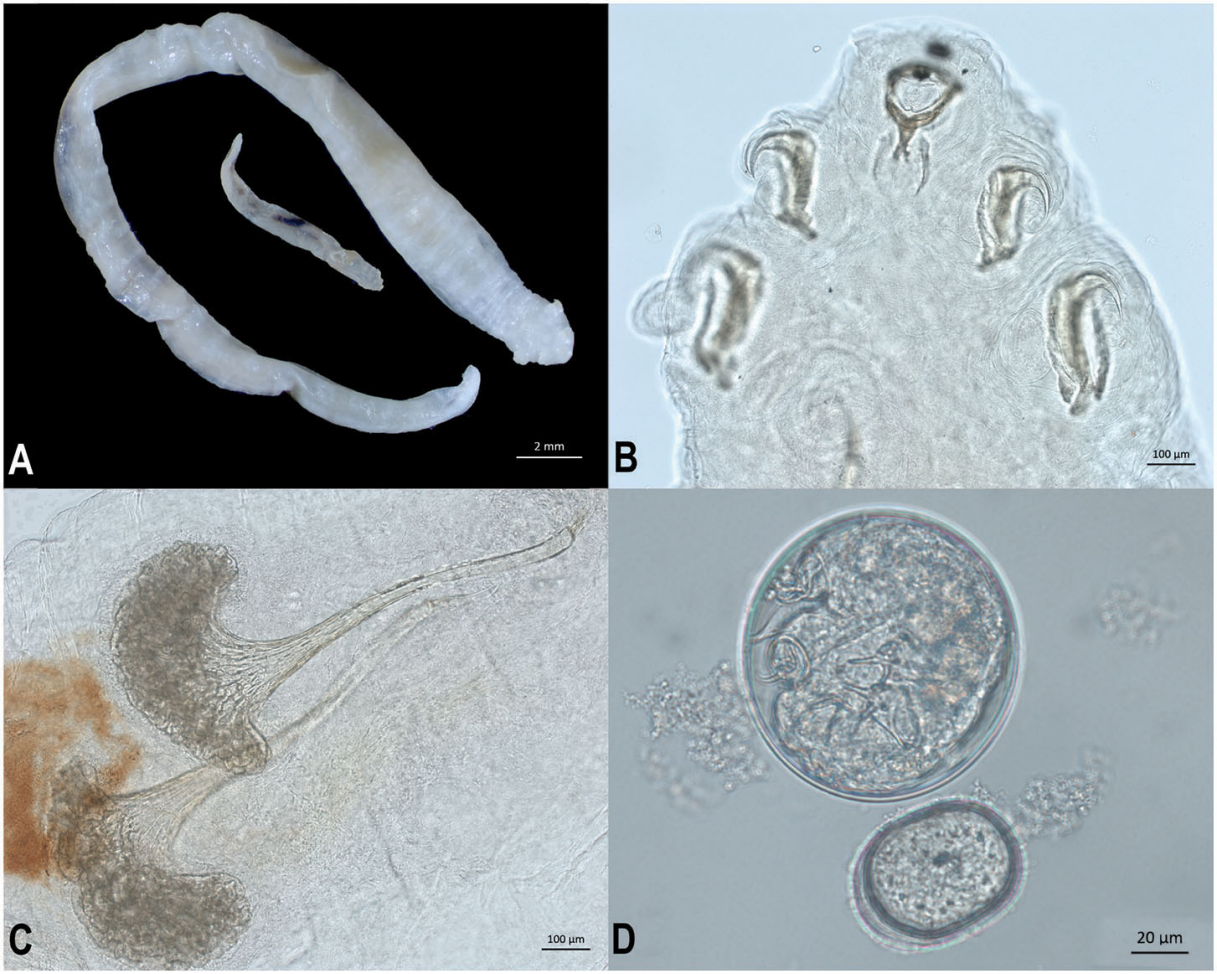
Life stages and morphological characteristics of *Raillietiella orientalis* pentastomes recovered from the lungs of a banded water snake (*Nerodia fasciata*). **(A)** Ethanol-fixed male (smaller) and female (larger) *R. orientalis* adults. **(B)** Male *R. orientalis* anterior hooks and buccal cadre. **(C)** Spicules from male *R. orientalis*. **(D)** Egg recovered from uterus of gravid female *R. orientalis*.

Adult male and female pentastomes (five female, one male) were preserved in 70% ethanol and examined morphologically (Figure 3A). Pentastomes were cleared using lactophenol, and species were identified based on total body length as well as measurements and characteristics of the buccal cadre and hook (Figure 3B), cephalothorax, and male spicules (3). Female pentastomes ranged from 29 to 51 mm in length, the male was 10 mm long. Posterior AB hook measurement for the largest female was 308 microns, and posterior BC hook measurement was 487 microns. Male spicules consisted of a flared base, with a deeply grooved reticulated pattern (Figure 3C). Females were gravid, and eggs recovered from the uterus were ~96 × 100 μm (Figure 3D). Morphologic characteristics were consistent with *Raillietiella orientalis*. Oral trematodes (renifers) were also preserved in 70% ethanol and examined morphologically. Morphologic characteristics were consistent with an *Ochetosoma* species (data not shown).

To confirm the morphologic identification of the parasite, nucleic acids were extracted from an ethanol fixed female pentastome using a DNEasy kit (Qiagen, Valencia, CA), and an approximately 425 bp portion of the small subunit ribosomal RNA gene (18S SSU) was amplified as previously described (4). The PCR product was resolved in a 1.5% agarose gel. Bands were excised and purified using a QIAquick gel extraction kit (Qiagen). The product was sequenced directly in both directions by a commercial sequencing facility (Genewiz, South Plainfield, New Jersey, USA). Primer sequences were trimmed and compared to known sequences using the NCBI Basic Local Alignment Search Tool (5). The amplified 18S sequence was 100% identical to numerous *R. orientalis* isolates, including those previously identified in Florida and reported by Miller et al. (6) and Farrell et al. (7).

## Discussion

**Figure 4:**
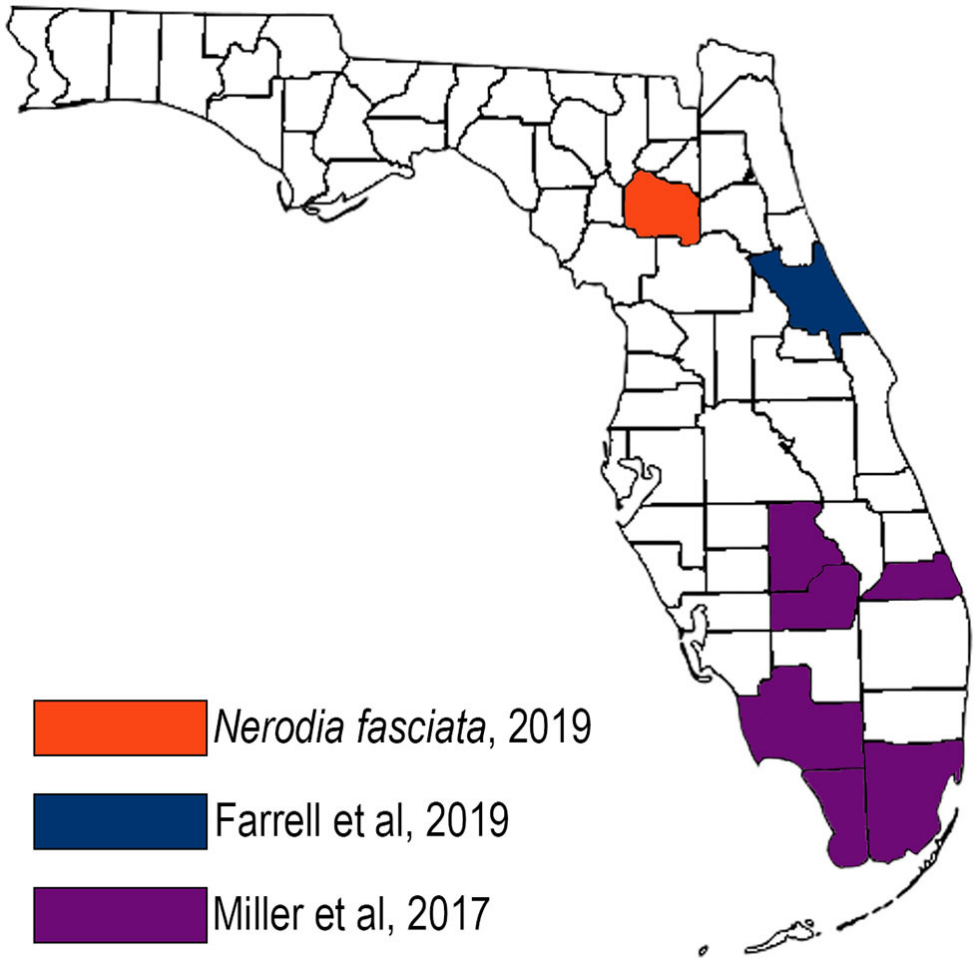
Map of *Raillietiella orientalis* documentations in Florida, United States showing the northward extension of the parasite over time.

This is the first report of *R. orientalis* in north central Florida. *Raillietiella orientalis* has been reported in several snake species in Asia and Australia, including those from the families Colubridae, Elaphidae, Viperidae, and Boidae (3, 8, 9). *Raillietiella orientalis* was first identified in the United States in 2017 in free-ranging, invasive Burmese pythons in the Florida Everglades as well as nine native snake species in six southern Florida counties (6) (Figure 4, purple counties). In that study, no parasites were identified during surveillance in northern Florida (including Alachua County) and southern Georgia. In 2018, *R. orientalis* were documented in three pygmy rattlesnakes (*Sistrurus miliarus*) more than 160 km north of the known range of Burmese pythons, in central Florida (7) (Figure 4, blue county). The spring 2019 identification of *R. orientalis* in a water snake in Alachua County demonstrates an additional range extension of nearly 150 km northward, and a total range extension of nearly 340 km beyond the northernmost range of the Burmese python.

This dramatic extension north suggests the potential of native or invasive species other than pythons serving as both successful intermediate and definitive hosts for the parasite. While the exact intermediate hosts of *R. orientalis* in Florida are unknown, most pentastome species use amphibians or reptiles as intermediate hosts, and definitive hosts for the many *Raillietiella* species include snakes and lizards (1, 8). The natural range of *Nerodia fasciata* is extensive, inhabiting coastal plains from southwest Alabama to North Carolina, along the Gulf States into Texas, and up the Mississippi River to Indiana (10). Along with sympatric species identified by Miller et al. (6) to be susceptible to infection, such as cottonmouths (*Agkistrodon piscivorus*), salt marsh snakes (*Nerodia clarkii*), garter snakes (*Thamnophis sirtalis*), and corn snakes (*Pantherophis guttatus*), there is a substantial risk that *Raillitiella orientalis* may spread throughout the southeastern United States, and potentially further.

The only named *Raillietiella* species for which sequence is available in public databases are *R. orientalis* and *R. indica*. Especially in light of the size variation seen in *R. orientalis*, the lack of reference sequence data for *Raillietiella* is concerning. Moreover, morphological features used for pentastomid identification may be altered by development in a different host (11). In the gecko species *Hemidactylus frenatus*, the species *Raillietiella indica* (syn *R*. *frenatus, R*. *hebitihamata*) develops hooks that are significantly different from those that *R. indica* develops in the marine toad (*Rhinella marina*), to the extent that they would morphologically identified as different species (11). Similarly, *R. indica* hook measurements vary significantly depending on whether the host is a Mediterranean gecko or a green anole (*Anolis carolinensis*) (12). Feral populations of another member of the gecko genus *Hemidactylus, H. turcicus* (Mediterranean gecko) populations are well established in Alachua County (13). *Raillietiella teagueselfi* has been reported from feral Mediterranean geckos in Texas, though this *Raillietiella* species lacks both reference sequence and molecular confirmation of a morphological diagnosis (14). Given the observed morphological plasticity of *Raillietiella* in different hosts, both morphological and genetic identification of *Raillietiella* species in different host species are warranted for accurate identification.

The spread of this parasite into definitive hosts in northern Florida suggests infection in competent squamate hosts in bordering southeastern states may be imminent. *Raillietiella orientalis* is not the only invasive *Raillietiella* species spreading through the continental US from invasive to native reptile species. *Raillietiella indica* was reported in the Mediterranean gecko, *Hemidactylus turcicus* in Texas in the early 1980s (15). Sakla et al. (12) later reported *R. indica* was not only infecting *H. turcicus*, but that it had also made its way into the native *Anolis carolinensis* in Louisiana, and noted an unpublished report of *R. indica* in *H. frenatus* in New Orleans, Louisiana as early as 2008. The authors speculated the introduction of *R. indica* into Louisiana could be from the Mediterranean gecko, known to New Orleans since 1949, or introduced from the Cuban brown anole, *Anolis sagrei*, another known host of *R. indica* (12). The exact origin of free-ranging Florida Burmese pythons, the natural host(s) for *R. orientalis*, in southern Florida is unknown; however, a prevailing theory is due to the release of animals destined for the pet trade in the 1990s. Although Burmese python sightings date back to approximately 1980, there has been a marked growth in python populations in the Everglades in the past 20 years. This population growth has been accompanied by severe declines in native mammals (16). The introduction of *R. orientalis* into Florida and ultimately, native Florida snakes, most likely occurred with the release of the Burmese pythons in southern Florida. No other reported *Raillietiella* hosts are native to North America or have been previously been reported in Florida (8). This case report highlights that parasites accompanying invasive species can also have a negative impact on native wildlife that can extend far beyond the range of the invasive host.

While *R. orientalis* is of limited clinical significance to the Burmese python, these invasive pentastomes are likely contributing to morbidity and mortality in native snake species (6, 7). Morbidity and mortality associated with these parasitic infections may be due to additional co-infections. Conversely, as North American snakes species have not coevolved with *R. orientalis*, the pathogenic effects may be more severe in a naïve host (17). Interestingly, this water snake had concurrent ophidiomycosis (snake fungal disease). Co-morbidities of ophidiomycosis and raillietielliasis are a significant cause of morbidity and mortality in pygmy rattlesnakes in central Florida (7). The role these two agents play in more widespread wild snake health in general warrants investigation. Moreover, as little is known about the life cycle of R. orientalis, including both the number of and species of viable intermediate hosts as well as the prepatent period, additional studies on the life cycle of *R. orientalis* are also needed.

## Data Availability Statement

All datasets generated for this study are included in the article/supplementary material.

## Ethics Statement

Ethical review and approval was not required for the animal study because care, until natural death, of the snake was provided in accordance to current standards of care. The snake was not a research snake and was found in the wild and cared for by UFCVM veterinarians.

## Author Contributions

HW contributed to gross morphological parasite identification and photographs. RO and MI contributed to postmortem examination and interpretation and photographs. AC, JW, and RO contributed to molecular parasite characterization. All authors contributed to the article and approved the submitted version.

## Conflict of Interest

The authors declare that the research was conducted in the absence of any commercial or financial relationships that could be construed as a potential conflict of interest.
